# Does the nicotine metabolite ratio moderate smoking cessation treatment outcomes in real‐world settings? A prospective study

**DOI:** 10.1111/add.14450

**Published:** 2018-10-30

**Authors:** Lion Shahab, Linda Bauld, Ann McNeill, Rachel F. Tyndale

**Affiliations:** ^1^ Department of Behavioural Science and Health University College London London UK; ^2^ UK Centre for Tobacco and Alcohol Studies Nottingham UK; ^3^ Usher Institute of Population Health Sciences and Informatics University of Edinburgh Edinburgh UK; ^4^ Addictions Department Institute of Psychiatry, Psychology and Neuroscience, King's College London London UK; ^5^ Campbell Family Mental Health Research Institute, Centre for Addiction and Mental Health (CAMH) and Departments of Psychiatry and Pharmacology and Toxicology University of Toronto Toronto Ontario Canada

**Keywords:** Nicotine metabolism, nicotine replacement therapy, pharmacogenomics, smoking, smoking cessation, stop smoking services, varenicline

## Abstract

**Background and aims:**

In smoking treatment trials comparing varenicline with transdermal nicotine replacement therapy (NRT), stratified by nicotine metabolite (3‐hydroxycotinine/cotinine) ratio (NMR), the relative benefit of varenicline is greater among normal rather than slow metabolizers. This study tested if the relative effectiveness of varenicline and NRT is associated with NMR status in a natural treatment setting. A secondary aim was to test if this relationship is moderated by behavioural support.

**Design:**

Prospective observational multi‐centre study with 4‐week and 52‐week follow‐up.

**Setting:**

Nine English Stop Smoking Services (SSS).

**Participants:**

Data came from 1556 smokers (aged ≥ 16 years) attending SSS between March 2012 and March 2013.

**Interventions:**

Participants received pharmacotherapy together with behavioural support.

**Measurements:**

The primary outcome was carbon monoxide‐verified continuous abstinence at both follow‐up times. Main explanatory variables were (1) NMR status [slow (NMR < 0.31, *n* = 451) versus normal (NMR ≥ 0.31, *n* = 1105) metabolizers]; (2) pharmacotherapy (varenicline versus NRT) and (3) behavioural support (individual versus group‐based treatment). Analyses adjusted for baseline socio‐demographic, SSS, mental/physical health and smoking characteristics.

**Findings:**

Of participants, 44.2% [95% confidence interval (CI) = 41.7–46.6%] and 8.0% (95% CI = 6.8–9.5%) were continuously abstinent at 4 and 52 weeks. Varenicline was more effective than NRT at 4 weeks (*P* < 0.001) but only marginally so at 52 weeks (*P* = 0.061). There was no or inclusive evidence that NMR status moderated relative efficacy of varenicline and NRT at 4‐ [*P* = 0.60, Bayes factor (BF) = 0.25] or 52‐week follow‐ups (*P* = 0.74, BF = 0.73). However, this relationship was moderated by behavioural support (*p* = 0.012): the relative benefit of varenicline over NRT at 52‐week follow‐up was greater in slow, not normal, metabolizers receiving group rather than individual support (*P* = 0.012).

**Conclusions:**

In a real‐world setting, the nicotine metabolite ratio status of treatment‐seeking smokers does not appear to contribute substantially to the differential effectiveness of varenicline and nicotine replacement therapy in Stop Smoking Services, when both pharmacotherapy and behavioural support are self‐selected.

## Introduction

Despite the existence of effective behavioural and pharmacological smoking cessation interventions, most treatment‐seeking smokers will still fail even with additional support 1, 2, 3, 4, 5, 6, 7. Given our increasing understanding of the molecular genetics of smoking and evidence of substantial heritability for tobacco addiction 8, 9, one option to improve cessation rates is to prescribe pharmacological treatment on the basis of genetically informed biomarkers 10, 11. The rationale is that the same genetic factors which predispose an individual to nicotine addiction may also moderate the response to pharmacotherapy 12. One such candidate biomarker is the nicotine metabolite ratio (NMR), calculated as the quotient of two major metabolites of nicotine [3′hydroxycotinine (3HC) and cotinine], which functions as a phenotypical surrogate of nicotine clearance 13.

The liver enzyme CYP2A6, part of the cytochrome P450 enzyme system, is largely responsible for the metabolism of nicotine into cotinine 14, and exclusively responsible for cotinine's metabolism into 3HC 15. The encoding gene *CYP2A6* is highly polymorphic, and has been associated with nicotine dependence and smoking behaviour 16, 17. However, as a phenotypical marker, NMR has an advantage over genotypical markers by incorporating genetic, environmental and demographic influences on nicotine metabolism 18, 19. It can also be measured easily and non‐invasively from saliva and urine as well as blood 20. Both NMR and categorized NMR (into slow versus normal/fast metabolizers) have been shown to be stable over time in *ad‐libitum* 21, 22 and treatment‐seeking smokers 23, and independent of smoking patterns and time since last cigarette, given the comparatively long half‐lives of cotinine and 3HC 13, 21. The NMR appears suitable for one‐time assessments, correlates well with clearance of nicotine that is administered orally or intravenously 13, 24 and is not affected by time of sampling 21, 25. Although NMR varies somewhat with sex 26, race 27, age 28 and body mass index 22, it is relatively consistent across different socio‐demographic and health characteristics, with such factors accounting for less than 8–9% in variance of NMR 19, 29, 30. However, NMR can be influenced by both environmental inducers and inhibitors, some of which can be transitory 19.

NMR is related to smoking behaviour in a number of ways. Smokers with a higher NMR, who therefore metabolize nicotine more quickly, also tend to be heavier smokers 31. In addition, faster metabolizers appear to smoke cigarettes more intensely, resulting in higher exposure to tobacco‐related carcinogens 32. However, the association of NMR with nicotine dependence and withdrawal symptoms is less clear‐cut, with some but not all studies finding an association of greater dependence and more severe withdrawal symptoms among faster metabolizers 20, 31. Similarly, data on the association of NMR with smoking cessation outcomes are mixed. Studies of pharmacological treatments have shown that slow metabolizers tend to have lower relapse rates than normal/fast metabolizers when treated with nicotine patch 33, 34 with placebo 35, or irrespective of treatment provided 36, whereas other studies have found an opposite pattern, with lower relapse rates among faster metabolizers using nicotine replacement therapy (with metabolism defined by genotype) 37 or when not using any treatment (with metabolism defined by NMR) 38. Still others find no difference in the effect of NMR on treatment with NRT but higher overall abstinence rates among slow metabolizers 39. Reflecting this uncertainty in the literature, a recent Cochrane review was inconclusive with regard to the superior efficacy of specific pharmacological treatment as a function of NMR 40.

The most rigorous assessment of the potential role of NMR for personalizing pharmacotherapy for smoking cessation comes from a recent placebo‐controlled clinical trial which prospectively randomized to treatment arm (varenicline or NRT patch) by NMR stratification 41. Clinical trials directly comparing varenicline with NRT have shown that varenicline is generally more effective than NRT 42. In contrast, this study found a significant NMR × treatment interaction, suggesting that varenicline was relatively more effective than transdermal NRT only for normal/fast (6‐month abstinence rates: 22 versus 13.6%) but not slow metabolizers (19.1 versus 21.6%). The implication is that in future normal/fast metabolizers should preferentially be prescribed varenicline and slow metabolizers transdermal NRT. However, given conflicting evidence to date and a call for replication and validation of NMR studies in different contexts 43, extension of these findings to other populations (treatment‐seeking smokers), different operationalizations of NMR and geographic locations is now required. This is particularly important as there are well‐known differences in the treatment provision and participant characteristics for clinical trials compared with general population studies 44, and consequent failures to replicate trial findings, e.g. for smoking cessation treatments 45, based on real‐world data. We have shown previously that the choice of pharmacotherapy in real‐world settings [Stop Smoking Services (SSS) in England] is not influenced by NMR status, suggesting that there is scope to optimize treatment allocation 30. However, in this context it is also important to consider other non‐pharmacological treatment factors, as the uptake of behavioural support was shown to differ as a function of NMR status, with normal metabolizers being less likely to choose group over individual support than slow metabolizers 30. The importance of this needs to be explored further.

In a large sample of treatment‐seekers in the United Kingdom, the present study therefore aimed to:

1. test whether NMR status (slow versus normal) moderates the short‐ and long‐term effectiveness of NRT compared with varenicline for smoking cessation in real‐world settings;
2. assess whether results are consistent across different operationalizations of NMR (as a continuous measure or based on quartiles) or when restricting pharmacotherapy to varenicline and transdermal nicotine patch alone; and
3. test whether this relationship is moderated by the type of behavioural treatment (individual or group support) received.

## Methods

### Design

This is a prospective observational study [Evaluating Long‐term Outcomes of NHS Stop Smoking Services (ELONS] carried out in nine SSS across three regions of England (North, South and Midlands). Participants were recruited into the study at their first visit, at which stage they provided saliva samples to determine NMR status, and were followed‐up via the SSS until 4 weeks post their quit date to determine short‐term continuous abstinence. Participants confirmed to be abstinent at 4‐week follow‐up were re‐contacted by the research team at 52 weeks post‐quit to determine long‐term continuous abstinence.

### Participants and procedure

The ELONS study recruited and consented 3044 participants, who were not pregnant and aged 16 or above, who accessed nine SSS in England between March 2012 and March 2013 and who set a firm quit date.

For the purpose of this analysis, participants who elected to not receive pharmacotherapy, only bupropion, or who chose combination therapy of NRT with varenicline or bupropion were excluded. Full details on ELONS methodology can be found elsewhere 46. Of the ELONS participants, 61.6% (*n* = 1875) agreed to provide saliva samples prior to start of treatment (44 samples of which were not useable and five were lost in the post; see 47 for details) and 51.1% (*n* = 1556) had complete baseline data and fulfilled inclusion and exclusion criteria, and thus constitute the analytical sample. In addition to providing saliva samples, participants also completed questionnaires to assess socio‐demographic, smoking, health‐related and treatment characteristics prior to start of treatment (questionnaires and anonymized data sets generated and/or analysed during the current study are available from the corresponding author on request). The study received ethical approval from the South East Scotland Research Ethics Committee (11/AL/0256) and from the University of Toronto. All participants included in this analysis consented to take part and research complied with the ethical principles on human research, according to the Declaration of Helsinki.

## Measures

### Outcome variables

#### Short‐ and long‐term continuous abstinence

Continuous abstinence was assessed at both 4‐ and 52‐week follow‐up post‐target quit dates 46. As per standard SSS criteria, abstinence at 4‐week follow‐up was defined as complete abstinence from smoking in the past 2 weeks, verified by an expired air‐carbon monoxide (CO) reading below 10 parts per million (p.p.m.), conducted at the SSS. As this study was interested in determining verified prolonged abstinence, only those participants who were defined as abstinent at 4‐week follow‐up were followed‐up further at 52 weeks. Smoking abstinence was again verified by CO reading, conducted at the participantss homes by the market research company TNS BMRB. Following recommended practice, participants lost to follow‐up were considered to be still smoking 48.

### Explanatory variables

#### Nicotine metabolite ratio

Saliva samples were collected with Sarstedt Salivettes^®^ and posted to University College London, where they were stored in −20°C freezers before being shipped to the University of Toronto or ABS laboratories for analysis. As earlier interlaboratory studies have shown comparable results among these different laboratories 49, 50, which was also the case in the current study (see 30 for details), analyses from both laboratories were pooled. As described previously 30, established liquid chromatography tandem mass spectrometry (LC–MS/MS) methodology with a 1 ng/ml limit of quantification (LOQ) 25, 50 was used to determine cotinine (COT) and trans‐3HC levels in saliva samples to calculate the NMR ratio (3HC/COT). Examination for analytical shift and reliability (conducted on 5% of samples) showed NMR results to be highly reliable (*R* ^2^ = 0.984) with no association between change in NMR and time between analyses (*R* ^2^ = 0.004). Given that NMR may be unstable for occasional and light smokers 51, samples with cotinine values below the standard cut‐off for smoking (10 ng/ml) were excluded. In cases where COT values were above 10 ng/ml but 3HC was below LOQ, the 3HC value was replaced by LOQ divided by the square root of two to compute the NMR 52. Based on population data from the previous prospectively NMR randomized clinical trial 41, participants in the analytical sample were classified into normal (NMR ≥ 0.31; *n* = 1105; 71.0%) or slow (NMR < 0.3; *n* = 451; 29.0%) metabolizer (see Table 1). Further information on socio‐demographic differences by NMR status has been published elsewhere 46.

**Table 1:** Sample characteristics by data availability.

All participants	Total sample (N = 3044)	Analytical sample (n = 1556)	Excluded sample (n = 1488)	P
Mean (SD) age	42.5 (14.1)	41.8 (14.1)	43.2 (14.1)	0.004
% (*n*) Female	55.9 (1701)	52.3 (814)	59.6 (887)	< 0.001
% (*n*) Higher SES (ABC1)	23.4 (712)	23.9 (372)	22.8 (340)	0.494
% (*n*) White	96.0 (2922)	94.9 (1477)	97.1 (1445)	0.002
% (*n*) Married/cohabiting	47.0 (1431)	46.7 (727)	47.3 (704)	0.771
% (*n*) Poor physical health	56.2 (1711)	56.0 (871)	56.5 (840)	0.798
% (*n*) Poor wellbeing	44.7 (1318)	43.1 (671)	46.5 (647)^a^	0.064
% (*n*) Higher dependence score (HSI ≥ 4)	49.4 (1489)	47.8 (743)	51.1 (746)^b^	0.068
% (*n*) Past‐year quit attempt	41.5 (1237)	40.9 (637)	42.0 (600)^c^^,^ d	0.552
% (*n*) Determination to quit				< 0.001
Not at all	8.8 (261)	8.4 (131)	9.1 (130)	
Very determined	39.5 (1176)	43.0 (669)	35.6 (507)	
Extremely determined	51.8 (1542)	48.6 (756)	55.2 (786)^e^	
% (*n*) Behavioural support				< 0.001
Individual support	78.6 (2385)	75.7 (1178)	81.7 (1207)	
Group support	21.4 (648)	24.3 (378)	18.3 (270)	
% (*n*) Pharmacological support				< 0.001
Single NRT^f^	17.7 (540)	17.2 (268)	18.3 (272)	
Combination NRT^g^	30.6 (933)	36.9 (574)	24.1 (359)	
Varenicline	43.0 (1308)	45.9 (714)	39.9 (594)	
Bupropion	0.9 (27)	–	1.8 (27)	
Varenicline and NRT	4.2 (129)	–	8.7 (129)	
Other combination	0.2 (5)	–	0.3 (5)	
None	3.4 (102)	–	6.9 (102)	
% (*n*) SSS Region				< 0.001
North	50.3 (1532)	44.9 (699)	56.0 (833)	
Midlands	38.3 (1166)	41.6 (647)	34.9 (519)	
South	11.4 (346)	13.5 (210)	9.1 (136)	
Participants with valid saliva sample	(*n* = 1826)	(*n* = 1556)	(*n* = 270)	
% (*n*) Slow metabolizers	28.5 (520)	29.0 (451)	25.6 (69)	0.273

SES = socio‐economic status; HSI = Heaviness of Smoking Index; NRT = nicotine replacement therapy; SSS = Stop Smoking Services; SD = standard deviation.

a 98 cases missing;

b 29 cases missing;

c 61 cases missing;

d 65 cases missing;

e 11 cases missing;

f single NRT products used were patches (*n* = 361; 66.9%), inhalator (*n* = 64; 11.9%), lozenges (*n* = 64; 11.9%), gum (*n* = 26; 4.8%), nasal/mouth spray (*n* = 23; 4.3%) and minitabs (n = 2; 0.4%);

g combination NRT most commonly involved patch together with inhalator (*n* = 289; 31.0%), lozenge (*n* = 182; 19.5%), nasal/mouth spray (*n* = 153; 16.4%) or gum (*n* = 85; 9.1%) and approximately 16% (*n* = 178) used more than two NRT products concurrently.

#### Pharmacotherapy and behavioural support characteristics

In this observational study, following consultation, participants chose their treatment freely, and this was recorded by SSS practitioners. Pharmacotherapy was dichotomized into varenicline or NRT product use (single or combined NRT). As indicated above, participants with combination non‐NRT/NRT treatment, bupropion or no pharmacotherapy were excluded from the analysis. The type of behavioural support chosen was also recorded as individual (one‐to‐one; non‐group drop‐in) versus group‐based (open/rolling groups; closed groups) support. Further information on socio‐demographic differences by treatment characteristics has been published elsewhere 46.

### Covariates

#### Socio‐demographic characteristics

Standard socio‐demographics [age, sex, socio‐economic status (SES), ethnicity, marital status] were recorded by SSS staff at baseline. SES was measured with the National Statistics Socio‐economic Classification (NSSEC) 53 and grouped into higher versus lower SES, using the NSSEC coding ABC1/C2DE (managerial occupations/manual and unemployed). Due to a relatively small number of participants with an ethnic minority background, ethnicity was split into ‘white British’ and ‘other’.

#### Stop Smoking Service and smoking characteristics

As local funding rules and policies are likely to affect treatment choice and success rates 54, SSS location was recorded and divided into North, Midlands and South regions of England. At baseline, nicotine dependence was measured with the Heaviness of Smoking Index (HSI) 55, classifying participants as having high (HSI score 4–6) or low dependence (HSI score 0–3) 56. Determination to quit was assessed on a one‐item four‐point Likert scale (ranging from ‘not at all determined’ to ‘extremely determined’), and whether participants had attempted to quit in the previous 12 months was also recorded (yes/no).

#### Health‐related characteristics

Participants were asked to provide information about any medical conditions, and those with at least one condition were coded as having poorer physical health compared with those without. Participants also completed the World Health Organization (WHO)‐5 wellbeing index 57, a tool used in primary care to determine psychological wellbeing using five questions scored from 0 to 5, with higher scores indicating better quality of life. Scores were summated and converted into a percentage of the maximum score 25, with scores ≤ 50% indicating low subjective wellbeing 58.

#### Analyses

In univariate analyses, group differences between the analytical and excluded samples in socio‐demographic, smoking, health‐related, NMR and treatment characteristics and smoking outcomes were assessed using χ^2^/*t*‐tests for categorical/continuous variables, respectively. Multivariable log‐binomial generalized linear models were used to provide risk ratios (RR). Analyses tested the independent relationships between smoking outcomes at 4‐week and 12‐month follow‐up and predictors, including a pharmacotherapy choice (NRT versus varenicline) by NMR status (slow versus normal NMR) interaction term to determine whether treatment effectiveness varied as a function of nicotine metabolism as well as their respective main effects, adjusting for covariates in Table 1. Age was transformed using the standard deviation of the variable as the scaling factor, as it did not meet linearity assumptions 59. Due to insufficient numbers, SSS location was not modelled as a random effect but included as a covariate in analysis. In sensitivity analyses, NMR status was defined based on quartiles to classify slow (1st quartile) versus fast (4th quartile) metabolizers or NMR was entered as a continuous variable. Bayes factors (BF) were also calculated for the primary analysis using standard cut‐offs to confirm findings and determine whether results can be interpreted as evidence to support the null‐hypothesis (BF < 1/3), the alternative hypothesis (BF > 3) or whether data were inclusive (BF < >1/3 and < 3). This was based on detecting an effect equivalent to the clinical trial data 41 using standard methodology with a half‐normal distribution and mean difference parameter estimates to represent the alternative hypothesis 60. Furthermore, given that previous clinical work had compared only the relative effectiveness of NRT patch versus varenicline among slow and normal metabolizers 41 the sample was restricted to users of these specific pharmacotherapies in sensitivity analysis. Lastly, as the uptake of group versus individual support has been shown to differ as a function of NMR status 30, a higher‐order interaction term (behavioural support × NMR status × pharmacotherapy) in addition to lower‐order interaction terms, and main effects was included in the main model to assess robustness of findings.

## Results

**Figure 1:**
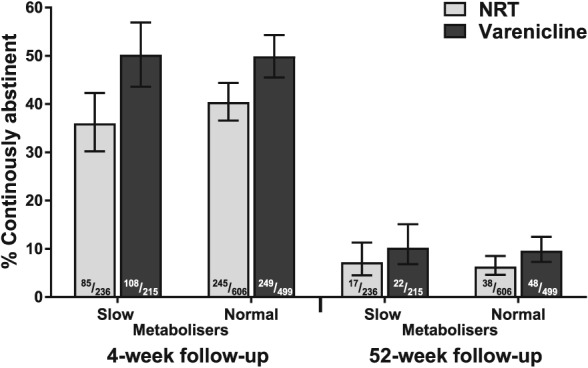
Raw continuous abstinence rates by pharmacotherapy type and nicotine metabolite ratio (NMR) status (n = 1556)

As shown in Table 1, the analytical sample (*n* = 1556) was somewhat younger, less likely to be female, white, to be extremely determined to quit or from the North of England than the excluded sample. They were also more likely to use group support. However, NMR status did not differ between those participants in the analytical and excluded samples who had provided saliva (Table 1). At 4‐week follow‐up, 44.2% [95% confidence interval (CI) = 41.7–46.6%] of participants were verified as continuously abstinent; this rate dropped to 8.0% (95% CI = 6.8–9.5%) at the 12‐month follow‐up. Figure 1 shows the raw abstinence rates broken down by type of pharmacotherapy used and NMR status (Supporting information, Fig. S1 presents adjusted data).

### Does NMR status moderate the short‐ and long‐term effectiveness of nicotine compared with non‐nicotine pharmacotherapy in SSS?

The effect of pharmacotherapy on outcomes did not appear to be moderated by NMR status. This was confirmed in adjusted analysis. There was no interaction of NMR status by pharmacotherapy choice on either 4‐ or 52‐week follow‐ups when controlling for all other variables (see Table 2). Bayes factors indicated that for the 4‐week follow‐up there was no effect (BF = 0.25) but that for the 52‐week follow‐up data were inconclusive (BF = 0.73). Given the lack of a support for the alternative hypothesis, the interaction term was therefore removed for the analyses below. Greater abstinence rates at 4‐ and 52‐week follow‐up were independently associated with lower dependence and being married or cohabiting. Higher socio‐economic status and use of varenicline were also associated with greater abstinence rates at 4‐week follow‐up but only marginally so at 52‐week follow‐up (Table 2). In addition, older age, greater determination to quit, using group support and attending SSS in the North or Midlands region of England were associated with greater abstinence rates only at 4‐week follow‐up.

**Table 2:** Associations between sample characteristics and smoking cessation outcomes at 4‐week and 12‐month follow‐up.

	Verified continuous abstinence (n = 1556)							
	4‐week follow‐up				12‐month follow‐up			
	Adj. RR (95% CI)	P	Adj. RR (95% CI)	P	Adj. RR (95% CI)	P	Adj. RR (95% CI)	P
NMR × pharmacotherapy (indicator: slow NMR and NRT)	0.88 (0.55–1.40)	0.586	–	–	1.15 (0.51–2.58)	0.741	–	–
Normal NMR (ref. slow NMR)	1.08 (0.78–1.49)	0.664	1.01 (0.80–1.28)	0.940	0.85 (0.46–1.56)	0.591	0.91 (0.61–1.37)	0.657
Varenicline (ref. NRT)	1.69 (1.14–2.51)	0.009	1.54 (1.24–1.91)	< 0.001	1.32 (0.67–2.58)	0.420	1.45 (0.99–2.12)	0.056
Group support (ref. individual support)	1.54 (1.17–2.02)	0.002	1.54 (1.17–2.03)	0.002	1.29 (0.82–2.04)	0.270	1.29 (0.81–2.04)	0.281
Age	1.50 (1.33–1.70)	< 0.001	1.50 (1.33–1.70)	< 0.001	1.15 (0.95–1.40)	0.143	1.16 (0.95–1.40)	0.144
Female (ref. male)	1.16 (0.93–1.43)	0.188	1.16 (0.93–1.43)	0.188	0.82 (0.56–1.20)	0.297	0.82 (0.56–1.20)	0.298
Higher SES/ABC1 (ref. C2DE)	1.43 (1.11–1.83)	0.005	1.43 (1.11–1.83)	0.005	1.43 (0.96–2.14)	0.081	1.43 (0.95–2.14)	0.083
White ethnicity (ref. other ethnicity)	0.98 (0.60–1.61)	0.938	0.99 (0.60–1.62)	0.963	1.19 (0.44–3.21)	0.737	1.18 (0.44–3.16)	0.746
Married/cohabiting (ref. single)	1.35 (1.09–1.67)	0.006	1.35 (1.09–1.67)	0.006	1.64 (1.11–2.41)	0.012	1.64 (1.11–2.41)	0.012
Poor physical health (ref. good physical health)	0.90 (0.71–1.13)	0.352	0.90 (0.71–1.13)	0.358	1.03 (0.68–1.56)	0.880	1.03 (0.68–1.56)	0.884
Poor wellbeing/WHO score ≤ 50% (ref. WHO score > 50%)	1.08 (0.87–1.35)	0.470	1.08 (0.87–1.35)	0.479	1.29 (0.88–1.89)	0.196	1.29 (0.88–1.90)	0.191
Higher dependence/HSI ≥ 4 (ref. HSI <4)	0.75 (0.60–0.93)	0.008	0.74 (0.60–0.92)	0.007	0.55 (0.38–0.81)	0.002	0.56 (0.38–0.81)	0.002
Determination to quit								
Very (ref. not determined)	1.73 (1.16–2.60)	0.008	1.73 (1.16–2.59)	0.008	0.80 (0.42–1.54)	0.512	0.81 (0.42–1.54)	0.514
Extremely (ref. not determined)	2.19 (1.47–3.28)	< 0.001	2.19 (1.47–3.27)	< 0.001	0.86 (0.45–1.64)	0.641	0.86 (0.45–1.64)	0.646
Past year quit attempt (ref. no attempt)	0.90 (0.73–1.12)	0.345	0.90 (0.73–1.12)	0.354	0.75 (0.51–1.12)	0.156	0.75 (0.51–1.11)	0.153
SSS region								
North (ref. South)	1.50 (1.06–2.11)	0.021	1.51 (1.07–2.13)	0.019	1.60 (0.82–3.14)	0.172	1.59 (0.81–3.13)	0.179
Midlands (ref. South)	1.65 (1.17–2.33)	0.005	1.66 (1.17–2.34)	0.004	1.85 (0.94–3.64)	0.077	1.84 (0.93–3.63)	0.079

Adj. RR = adjusted risk ratio (adjusted for all variables shown); CI = confidence interval; NMR = nicotine metabolite ratio; HSI = Heaviness of Smoking Index; SES = socio‐economic status; NRT = nicotine replacement therapy; SSS = Stop Smoking Services; WHO – World Health Organization; ref. = reference category.

### Are results consistent across different operationalizations of NMR or when restricting pharmacotherapy to varenicline and transdermal nicotine patch alone?

Further sensitivity analyses were conducted to assess the robustness of findings. Characterizing participants into slow versus fast metabolizers based on quartiles or using NMR as a continuous variable did not affect the observed associations, or lack thereof. Similarly, restricting the sample to NRT patch and varenicline users only did not alter results materially (see Supporting information, Tables S1–S3).

### Is the relationship between smoking cessation outcomes, NMR status and pharmacotherapy moderated by the type of behavioural treatment received?

**Figure 2:**
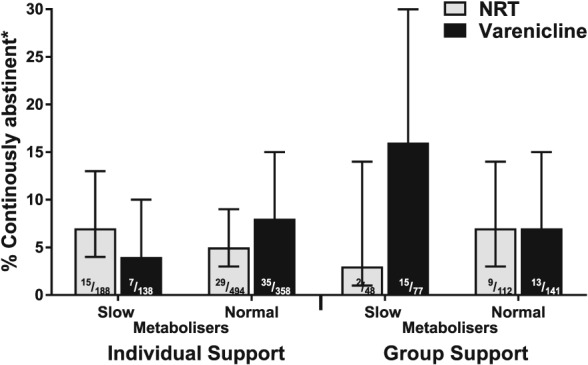
Adjusted continuous 52‐week verified abstinence rates based on estimated marginal means by pharmacotherapy and behavioural support type and nicotine metabolite ratio (NMR) status (n = 1556)

Lastly, a higher‐order interaction was included in the sensitivity analysis to determine whether or not the putative effect of NMR status on pharmacotherapy effectiveness is dependent on the type of behavioural support provided. This was considered, at least in part, as we had previously observed self‐selection of group support by NMR, where normal metabolizers were less likely to use group support 30. Behavioural support choice moderated the impact of the NMR status by pharmacotherapy choice relationship on the 52‐week (Wald χ^2^ = 6.33, *P* = 0.012) but not 4‐week follow‐up abstinence rates. As can be seen in Fig. 2, the relative benefit of varenicline over NRT at 52‐week follow‐up was greater in slow metabolizers receiving group rather than individual support (adjusted RR for interaction 11.3, 95% CI =1.76–71.7, *P* = 0.011), whereas this was not the case for normal metabolizers (adjusted RR for interaction 0.71, 95% CI = 0.25–2.02, *P* = 0.515). These results were not altered materially when including additional covariate–exposure interactions for two established determinants for smoking cessation, nicotine dependence and social grade 61.

## Discussion

This study set out to evaluate whether NMR status moderates the impact of NRT relative to varenicline on smoking cessation rates in a general population sample of treatment‐seeking smokers. Contrary to previous clinical work 41, we did not observe a benefit of varenicline over NRT for normal metabolizers compared with slow metabolizers. We also did not find any differences in abstinence rates between normal and slow metabolizers when controlling for other known confounders. In agreement with previous work 42, 61, 62, greater abstinence rates were associated with lower dependence and living together with a partner and, to a lesser degree, with social grade as well as treatment with varenicline rather than NRT. Short‐term abstinence only was also associated with older age, determination to quit and group rather than individual support, as has been previously shown 54, 63.

Several reasons may account for the failure to replicate clinical trial findings in this real‐world study. First, this may be due to differences in the socio‐demographic composition of the type of participants included in clinical trials and population studies. Clinical trials often exclude smokers with comorbidities such as mental health issues and may attract more proactive participants, motivated by financial remuneration. The NMR‐based clinical trial also excluded individuals taking drugs which were known inhibitors of CYP2A6, which could transiently (or longer) convert a normal metabolizer to a slow metabolizer 41. By contrast, our study passively recruited all smokers attending stop smoking services, who were not reimbursed for participation. Secondly, and relatedly, given ethnic variation in NMR 27, our results may reflect genuine differences in UK versus North American smokers, where the current trial was 95% white, which was substantially lower (55% white) in the North American trial 41. Thirdly, while clinical trials have high internal validity assessing efficacy of treatments with high fidelity and good implementation, they lack the external validity of population studies which assess treatment effectiveness outside a controlled environment, with suboptimal implementation. Fourthly, clinical trials will seek to maximize follow‐up response to obtain an accurate estimate of the treatment effect, whereas follow‐up rates in population studies such as ours tend to be lower, leading to potential underestimates of treatment effects in the context of intention‐to‐treat analysis. Fifthly, compliance was not assessed, and it is difficult to compare drug effects if compliance differs. The NRT arms differed substantially, with the majority of NRT users being dual users while the previous clinical trial used only transdermal patch. It is possible that dual NRT is as useful for normal metabolizers as varenicline. Lastly, and importantly, whereas in clinical trials participants are randomly allocated to treatment, smokers in our study self‐selected their treatment and normal metabolizers were less likely to use group support (see limitations below). Although we controlled for a range of covariates, the difference in this study and the previous clinical trial may in part, therefore, reflect confounding due to factors not accounted for.

While we did not detect the predicted interaction of NMR status with pharmacotherapy type on smoking cessation outcomes, we observed an association of NMR status with pharmacotherapy effectiveness as a function of the behavioural support provided. Specifically, the effectiveness of varenicline over NRT was markedly more pronounced in the context of group rather than individual behavioural support, but only for slow and not normal metabolizers. It should be acknowledged that this finding needs to be interpreted with caution, given small numbers. Individual support is more commonly accessed via community practitioners (e.g. general practitioners, pharmacists) who provide shorter and fewer counselling sessions, whereas group support is almost exclusively accessed via specialist stop smoking clinics which provide more intensive, longer treatment, often over six to eight face‐to‐face sessions 46. Given that varenicline has a worse side‐effect profile for slow rather than normal metabolizers 41, slow metabolizers may discontinue varenicline earlier in the context of individual support, with less intensive support and limited advice on medication adherence. By contrast, being provided with more extensive advice on medication side effects and the importance of adherence in the context of group support, slow metabolizers may be more likely to continue treatment with varenicline, resulting in superior outcomes. This would be less of an issue for normal metabolizers who experience fewer side‐effects.

This study has a number of limitations. As previously mentioned, participants self‐selected their treatment, and thus findings may be the result of an artefact due to confounding. Although we controlled for a range of potentially important covariates, not all putative factors (including medication adherence) were measured and some variables were only assessed with a single item. In particular, as reported previously, normal metabolizers were less likely to choose group behavioural support 30, which appears to affect pharmacotherapy outcome in this analysis. While the longitudinal design allowed us to investigate temporal effects, it resulted in high levels of attrition and relatively small numbers of smokers who had quit by the end of the study, limiting our power to detect more complex effects. Moreover, even though we used a prospective design, this does not allow us to make causal claims. Finally, while the initial sample collected was largely representative of smokers seeking treatment in the United Kingdom, there were some marked demographic and treatment differences between those who had complete data and were included in the analysis and those who were excluded. Findings may therefore not generalize beyond the current sample.

## Conclusions

Our study did not replicate clinical trial data, suggesting that NMR status of treatment‐seeking smokers does not contribute substantially to differential pharmacotherapy effectiveness in Stop Smoking Services, when both pharmacotherapy and behavioural support are self‐selected. While there may be a number of reasons for this, one potential explanation for this finding is the distinctly different effect that varying levels of behavioural support may have on treatment with NRT and varenicline for slow versus normal metabolizers. If correct, and corroborated by further studies, this interpretation would have clear implications for treatment delivery to slow and normal metabolizers: the benefits of varenicline over NRT previously identified may only become apparent for slow metabolizers if sufficient behavioural support is provided. Altogether, our results suggest that NMR status may not have a large effect on real‐world self‐selected treatment outcomes and that the impact of NMR may be context‐dependent. While this suggests one potential reason for the apparent discordance in the literature, further clarification of the role of the rate of nicotine metabolism, choice of group counselling and their interaction with treatment effect is required. Specifically, it will be important to understand (1) whether dual NRT behaves in the same way as transdermal NRT alone with respect to NMR predicting outcomes in the context of clinical trials and (2) the impact of the type of counselling (and associated treatment adherence) on NMR status by pharmacotherapy effects in the context of real‐world settings where treatment choice is based on NMR status, which is prescriber‐selected rather than self‐selected.

## Declaration of interests

L.S. has received honoraria for talks, an unrestricted research grant and travel expenses to attend meetings and workshops from Pfizer and an honorarium to sit on advisory panel from Johnson&Johnson, both pharmaceutical companies that make smoking cessation products. He has acted as paid reviewer for grant awarding bodies and as a paid consultant for health care companies. Other research has been funded by the government, a community‐interested company (National Centre for Smoking Cessation) and charitable sources. He has never received personal fees or research funding of any kind from alcohol, electronic cigarette or tobacco companies. R.T. has consulted for Apotex and Quinn Emanuel on topics unrelated to smoking or NMR. The other authors have no conflicts of interest.

## Supporting information


**Figure S1** Adjusted continuous abstinence rates by pharmacotherapy type and NMR status (N = 1556). Error bars show 95% confidence intervals; NMR – Nicotine metabolite ratio; NRT – Nicotine replacement therapy; Numbers in bars represent adjusted n/N; *Estimated marginal means, controlling for other covariates
**Table S1** Associations between sample characteristics and smoking cessation outcomes at 4‐week and 12‐month follow‐up with NMR status based on quartiles
**Table S2** Associations between sample characteristics and smoking cessation outcomes at 4‐week and 12‐month follow‐up with continuous NMR
**Table S3** Associations between sample characteristics and smoking cessation outcomes at 4‐week and 12‐month follow‐up, restricted to nicotine patch and varenicline users
