# Supplementary material for: Does the nicotine metabolite ratio moderate smoking cessation treatment outcomes in real‐world settings? A prospective study
Source: Addiction. 2018 Oct 30;114(2):304–14. doi: 10.1111/add.14450 (PMC6492100; doi:10.1111/add.14450)
Supplement: Supplementary file 1 — Figure S1 Adjusted continuous abstinence rates by pharmacotherapy type and NMR status (N = 1556). Error bars show 95% confidence intervals; NMR – Nicotine metabolite ratio; NRT – Nicotine replacement therapy; Numbers in bars represent adjusted n/N; *Estimated marginal means, controlling for other covariates Table S1 Associations between sample characteristics and smoking cessation outcomes at 4‐week and 12‐month follow‐up with NMR status based on quartiles Table S2 Associations between sample characteristics and smoking cessation outcomes at 4‐week and 12‐month follow‐up with continuous NMR Table S3 Associations between sample characteristics and smoking cessation outcomes at 4‐week and 12‐month follow‐up, restricted to nicotine patch and varenicline users [file ADD-114-304-s001.docx]

**Supplementary Tables and Figures**

**Figure legends**

Figure S1: Error bars show 95% confidence intervals; NMR – Nicotine metabolite ratio; NRT – Nicotine replacement therapy; Numbers in bars represent adjusted n/N; *Estimated marginal means, controlling for other covariates

**Supplementary Tables**

Supplementary Table S1: Associations between sample characteristics and smoking cessation outcomes at 4-week and 12-month follow-up with NMR status based on quartiles

|  | **Verified continuous abstinence**  (N=781) | | | |
| --- | --- | --- | --- | --- |
|  | **4-week follow-up** | | **12-month follow-up** | |
|  | Adj. RR (95%CI) | *P* | Adj. RR (95%CI) | *P* |
| **NMR quartile x Pharmacotherapy** (indicator: first quartile and NRT) | 0.80 (0.44-1.47) | 0.474 | 1.06 (0.38-2.93) | 0.913 |
| **Fourth NMR quartile** (ref. first quartile) | 1.04 (0.68-1.59) | 0.871 | 0.87 (0.41-1.84) | 0.707 |
| **Varenicline** (ref. NRT) | 1.70 (1.10-2.62) | 0.017 | 1.30 (0.65-2.60) | 0.467 |
| **Group support** (ref. individual support) | 1.56 (1.06-2.30) | 0.024 | 2.08 (1.12-3.89) | 0.020 |
| **Age** | 1.55 (1.30-1.84) | <0.001 | 1.16 (0.90-1.49) | 0.250 |
| **Female** (ref. male) | 1.17 (0.85-1.60) | 0.332 | 1.07 (0.64-1.79) | 0.805 |
| **Higher SES/ ABC1** (ref. C2DE) | 1.49 (1.04-2.12) | 0.029 | 1.26 (0.71-2.23) | 0.422 |
| **White ethnicity** (ref. other ethnicity) | 1.02 (0.50-2.09) | 0.949 | 1.67 (0.35-7.97) | 0.519 |
| **Married/Cohabiting** (ref. single) | 1.42 (1.05-1.93) | 0.025 | 1.67 (0.99-2.82) | 0.057 |
| **Poor physical health** (ref. good physical health) | 1.04 (0.74-1.45) | 0.823 | 1.03 (0.57-1.87) | 0.919 |
| **Poor wellbeing/WHO score ≤50%** (ref. WHO score >50%) | 1.17 (0.86-1.61) | 0.322 | 1.30 (0.75-2.24) | 0.347 |
| **Higher dependence/HSI≥ 4** (ref. HSI <4) | 0.77 (0.57-1.05) | 0.099 | 0.51 (0.30-0.87) | 0.014 |
| **Determination to quit**  Very (ref. not determined)  Extremely (ref. not determined) | 1.66 (0.92-3.00)  2.04 (1.13-3.69) | 0.093  0.018 | 1.07 (0.39-2.99)  1.28 (0.46-3.57) | 0.893  0.637 |
| **Past year quit attempt** (ref. no attempt) | 0.88 (0.65-1.19) | 0.407 | 0.60 (0.35-1.04) | 0.068 |
| **SSS Region**  North (ref. South)  Midlands (ref. South) | 1.67 (1.03-2.72)  1.94 (1.19-3.16) | 0.039  0.008 | 1.43 (0.59-3.46)  1.36 (0.57-3.25) | 0.423  0.496 |

Adj. RR = adjusted risk ratio (adjusted for all variables shown); CI = confidence interval; NMR = nicotine metabolite ratio; HSI = Heaviness of Smoking Index; SES = socioeconomic status; NRT = nicotine replacement therapy; SSS = Stop smoking services; WHO – World Health Organisation; ref. = reference category

Supplementary Table S2: Associations between sample characteristics and smoking cessation outcomes at 4-week and 12-month follow-up with continuous NMR

|  | **Verified continuous abstinence**  (N=1,556) | | | |
| --- | --- | --- | --- | --- |
|  | **4-week follow-up** | | **12-month follow-up** | |
|  | Adj. RR (95%CI) | *P* | Adj. RR (95%CI) | *P* |
| **Continuous NMR x Pharmacotherapy** (indicator: NRT) | 0.73 (0.36-1.48) | 0.380 | 1.27 (0.36-4.51) | 0.717 |
| **Continuous NMR** | 1.27 (0.81-2.00) | 0.298 | 0.83 (0.34-1.99) | 0.674 |
| **Varenicline/bupropion** (ref. NRT) | 1.79 (1.20-2.69) | 0.005 | 1.30 (0.65-2.61) | 0.463 |
| **Group support** (ref. individual support) | 1.54 (1.17-2.02) | 0.002 | 1.30 (0.82-2.05) | 0.262 |
| **Age** | 1.49 (1.32-1.69) | <0.001 | 1.15 (0.95-1.40) | 0.150 |
| **Female** (ref. male) | 1.15 (0.93-1.43) | 0.200 | 0.81 (0.56-1.19) | 0.290 |
| **Higher SES/ ABC1** (ref. C2DE) | 1.43 (1.12-1.84) | 0.005 | 1.43 (0.96-2.14) | 0.082 |
| **White ethnicity** (ref. other ethnicity) | 0.97 (0.59-1.59) | 0.903 | 1.18 (0.44-3.17) | 0.737 |
| **Married/Cohabiting** (ref. single) | 1.35 (1.09-1.67) | 0.006 | 1.64 (1.11-2.41) | 0.013 |
| **Poor physical health** (ref. good physical health) | 0.89 (0.71-1.12) | 0.331 | 1.03 (0.68-1.56) | 0.888 |
| **Poor wellbeing/WHO score ≤50%** (ref. WHO score >50%) | 1.09 (0.87-1.35) | 0.454 | 1.29 (0.88-1.89) | 0.192 |
| **Higher dependence/HSI≥ 4** (ref. HSI <4) | 0.74 (0.60-0.92) | 0.007 | 0.55 (0.38-0.81) | 0.002 |
| **Determination to quit**  Very (ref. not determined)  Extremely (ref. not determined) | 1.72 (1.15-2.58)  2.19 (1.46-3.27) | 0.008  <0.001 | 0.81 (0.42-1.55)  0.86 (0.45-1.64) | 0.517  0.648 |
| **Past year quit attempt** (ref. no attempt) | 0.90 (0.72-1.12) | 0.333 | 0.75 (0.51-1.12) | 0.157 |
| **SSS Region**  North (ref. South)  Midlands (ref. South) | 1.51 (1.07-2.13)  1.66 (1.17-2.34) | 0.019  0.004 | 1.60 (0.82-3.13)  1.84 (0.93-3.63) | 0.171  0.078 |

Adj. RR = adjusted risk ratio (adjusted for all variables shown); CI = confidence interval; NMR = nicotine metabolite ratio; HSI = Heaviness of Smoking Index; SES = socioeconomic status; NRT = nicotine replacement therapy; SSS = Stop smoking services; WHO – World Health Organisation; ref. = reference category

Supplementary Table S3: Associations between sample characteristics and smoking cessation outcomes at 4-week and 12-month follow-up, restricted to nicotine patch and varenicline users

|  | **Verified continuous abstinence**  (N=899) | | | |
| --- | --- | --- | --- | --- |
|  | **4-week follow-up** | | **12-month follow-up** | |
|  | Adj. RR (95%CI) | *P* | Adj. RR (95%CI) | *P* |
| **NMR x Pharmacotherapy** (indicator: slow NMR and NRT patch) | 0.76 (0.36-1.64) | 0.490 | 0.36 (0.04-3.42) | 0.376 |
| **Normal NMR** (ref. slow NMR) | 1.30 (0.65-2.62) | 0.456 | 2.88 (0.32-25.6) | 0.343 |
| **Varenicline** (ref. NRT patch) | 1.70 (0.88-3.28) | 0.115 | 6.25 (0.77-50.7) | 0.086 |
| **Group support** (ref. individual support) | 1.80 (1.27-2.53) | 0.001 | 1.74 (1.02-3.00) | 0.044 |
| **Age** | 1.34 (1.13-1.57) | 0.001 | 1.19 (0.93-1.53) | 0.166 |
| **Female** (ref. male) | 1.13 (0.92-1.40) | 0.142 | 0.67 (0.42-1.10) | 0.111 |
| **Higher SES/ ABC1** (ref. C2DE) | 1.30 (0.95-1.79) | 0.100 | 1.64 (1.00-2.70) | 0.051 |
| **White ethnicity** (ref. other ethnicity) | 0.37 (0.12-0.98) | 0.047 | 1.41 (0.16-12.5) | 0.760 |
| **Married/Cohabiting** (ref. single) | 1.35 (1.02-1.79) | 0.039 | 1.78 (1.07-2.96) | 0.026 |
| **Poor physical health** (ref. good physical health) | 0.89 (0.67-1.19) | 0.424 | 0.82 (0.48-1.41) | 0.478 |
| **Poor wellbeing/WHO score ≤50%** (ref. WHO score >50%) | 1.18 (0.89-1.56) | 0.264 | 1.00 (0.61-1.65) | 0.999 |
| **Higher dependence/HSI≥ 4** (ref. HSI <4) | 0.70 (0.52-0.93) | 0.014 | 0.60 (0.36-0.99) | 0.047 |
| **Determination to quit**  Very (ref. not determined)  Extremely (ref. not determined) | 1.77 (1.08-2.90)  1.93 (1.19-3.12) | 0.023  0.008 | 0.62 (0.28-1.40)  0.74 (0.34-1.61) | 0.249  0.447 |
| **Past year quit attempt** (ref. no attempt) | 0.85 (0.64-1.12) | 0.249 | 0.74 (0.45-1.22) | 0.231 |
| **SSS Region**  North (ref. South)  Midlands (ref. South) | 1.47 (0.92-2.34)  1.88 (1.18-3.01) | 0.110  0.008 | 2.65 (1.03-6.84)  2.26 (0.83-6.17) | 0.044  0.112 |

Adj. RR = adjusted risk ratio (adjusted for all variables shown); CI = confidence interval; NMR = nicotine metabolite ratio; HSI = Heaviness of Smoking Index; SES = socioeconomic status; NRT = nicotine replacement therapy; SSS = Stop smoking services; WHO – World Health Organisation; ref. = reference category

**Supplementary Figures**

Figure S1: Adjusted continuous abstinence rates by pharmacotherapy type and NMR status (N=1,556)
